# Objective Error Criterion for Evaluation of Mapping Accuracy Based on Sensor Time-of-Flight Measurements

**DOI:** 10.3390/s8128248

**Published:** 2008-12-15

**Authors:** Billur Barshan

**Affiliations:** Department of Electrical and Electronics Engineering, Bilkent University, Bilkent 06800 Ankara, Turkey; Tel: +90 312 290 2161; Fax: +90 312 266 4192; E-mail: billur@ee.bilkent.edu.tr URL: http://www.ee.bilkent.edu.tr/∼billur

**Keywords:** mapping, map errors, error criterion, range sensing, time-of-flight measurements, ultrasonic sensing, laser range finders, autonomous robots, Hausdorff metric, median error

## Abstract

An objective error criterion is proposed for evaluating the accuracy of maps of unknown environments acquired by making range measurements with different sensing modalities and processing them with different techniques. The criterion can also be used for the assessment of goodness of fit of curves or shapes fitted to map points. A demonstrative example from ultrasonic mapping is given based on experimentally acquired time-of-flight measurements and compared with a very accurate laser map, considered as absolute reference. The results of the proposed criterion are compared with the Hausdorff metric and the median error criterion results. The error criterion is sufficiently general and flexible that it can be applied to discrete point maps acquired with other mapping techniques and sensing modalities as well.

## 1. Introduction

A truly autonomous robot should be able to build an accurate spatial model of its unknown physical environment based on the sensory data it acquires. This problem is addressed by the field of robotic mapping. The environment in which the robot operates could be a static one, or more realistically, a dynamically changing environment. Therefore, the selection of a suitable mapping scheme is an important task [1]. The mapping approaches used in the 1980s and the early 1990s can be categorized into two, as metric and topological approaches. Metric maps capture the geometric properties of the environment, whereas topological maps describe the connectivity of its different parts. Since the 1990s, probabilistic techniques have dominated the field of robotic mapping. In particular, the simultaneous localization and mapping (SLAM) problem, also referred as concurrent mapping and localization (CML), has received considerable interest and has been a highly active research area over the past two decades. Consequently, a large family of algorithms for concurrent estimation of the map and the robot pose have emerged [2, 3].

Since robot mapping is characterized by sensor measurement noise and uncertainty, most of the current robotic mapping algorithms in the literature are probabilistic in nature [4]. These algorithms employ probabilistic models of the robot and its surroundings and rely on probabilistic inference to extract the map of the environment based on sensory measurements. The different approaches can be classified as Kalman filtering techniques [5–7], approaches based on Dempster's expectation-maximization algorithm [8, 9], occupancy grid techniques [10, 11], techniques for learning object models or features in the environment [12–14], and hybrid approaches [15].

Feature-based approaches are based on extracting the geometry of the environment from sensor data as the first step in data interpretation process (e.g., edge detection, fitting straight-lines or curves to obstacle boundaries, differentiating features such as planar walls, corners, and edges, open and closed doors, and corridors). Important issues to consider are the representation of uncertainty, suitability of the selected feature to the environment and the type of data, the reliability of the feature extraction process, and the speed with which the model can be constructed. An alternative representation is the *certainty* or *occupancy grid* where the environment is discretized into an array of cells. The difficulty of line segment-based feature extraction has been an important factor in the development of the grid concept proposed by Elfes [10, 11]. [Reference 1] provides a comprehensive survey and further references on robotic mapping, with a focus on indoor environments.

The map of an unknown environment can be acquired by making range measurements with a variety of sensing modalities, such as ultrasonic sensors, laser range finders, or millimeter wave radar. However, the acquired map is usually erroneous, consisting of outliers, gaps, noisy or incorrect measurements. Some of the measurements are caused by multiple or higher-order reflections from specular (mirror-like) surfaces, or crosstalk between multiple sensors, making the data difficult to interpret. This is particularly a problem with airborne ultrasonics and underwater sonar sensing. Researchers have identified regions of constant depth (RCDs) [16, 17], and defined and processed ultrasonic arc maps that represent the angular uncertainty of ultrasonic sensors [18–20]. Line segments [21], polynomials [22], and snake curves [23] have been fitted to acquired ultrasonic maps to represent the map points more compactly and efficiently. However, to the best of our knowledge, there is no well-defined objective criterion or systematic method for the evaluation of map accuracy. Lack of such criteria also makes it difficult to compare maps acquired by different mapping techniques and sensor types. Although the areas of sensing, measurement technology, and mapping have developed considerably and have been extended to 3-D over the recent years [9, 24–26], assessment of the accuracy of the acquired maps and comparison between different mapping techniques is an important issue not extensively studied. In most mapping studies, the map accuracy is assessed by graphically displaying the acquired map together with the true map, and a subjective, qualitative judgment is made by visual comparison. The main contribution of this paper is the proposition of an objective and quantitative error criterion for the accuracy assessment and comparative evaluation of acquired maps.

In Section 2., we give a description of the proposed error criterion and provide two other criteria for comparison: the Hausdorff metric and the median error. The use of the criterion is demonstrated through an example from ultrasonic and laser sensing in Section 3. Section 4. provides details of the experimental procedure and compares the results of the proposed criterion with the Hausdorff metric and the median error. Section 5. discusses the limiting circumstances for the criterion that may arise when there are temporal or spatial differences in acquiring the maps. The last section concludes the paper by indicating some potential application areas and providing directions for future research.

## 2. The Error Criterion

Let *P* ⊂ ℝ^3^ and *Q* ⊂ ℝ^3^ be two finite sets of arbitrary points with *N*_1_ points in set *P* and *N*_2_ points in set *Q*. We do not require the correspondence between the two sets of points to be known. Each point set could correspond to either (i) an acquired set of map points, (ii) discrete points corresponding to an absolute reference (the true map), or (iii) some curve (2-D) or shape (3-D) fit to the map points. The absolute reference could be an available true map or plan of the environment or could be acquired by making range or time-of-flight measurements through a very accurate sensing system.

The well-known Euclidean distance *d*(**p***_i_*, q*_j_*) : ℝ^3^ → ℝ^≥^^0^ of the *i*'th point in set *P* with position vector p*_i_* = (*p_xi_*, *p_yi_*, *p_zi_*)*^T^* to the *j*'th point q*_j_* = (*q_xj_*, *q_yj_*, *q_zj_*)*^T^* in set *Q* is given by:

(1) $$d({\text{p}}_{i},{\text{q}}_{j})=\sqrt{{\left(p_{xi}-q_{xj}\right)}^{2}+{\left(p_{yi}-q_{yj}\right)}^{2}+{\left(p_{zi}-q_{zj}\right)}^{2}}i\in 1,\ldots ,N_{1}\;j\in 1,\ldots ,N_{2}$$

There is a choice of metrics to measure the similarity between two sets of points, each with certain advantages and disadvantages:

A very simple metric is to take the minimum of the distances between any point of set *P* and any point of *Q*. This corresponds to a *minimin* function and is defined as:

(2) $$D(P,Q)=\underset{{\text{p}}_{i}\in P}{\min}\{\underset{{\text{q}}_{i}\in Q}{\min}\{d({\text{p}}_{i},{\text{q}}_{i})\}$$

In other words, for every point **p***_i_* of set *P*, we find its minimum distance to any point **q***_j_* of *Q* and we keep the minimum distance found among all points **p***_i_*. This metric has very low informative content since the minimum value is not representative of the whole point set. For the same value of *D*(*P*, *Q*), the shape, position, and orientation of the two point sets can be very different with respect to each other. Even if one single point of the two point sets overlap, the metric will take the value zero. Since this metric has obvious shortcomings, we did not not consider it for our purpose of comparing maps.

The error criterion we propose for measuring the closeness or similarity between sets *P* and *Q* overcomes the shortcomings of the minimin function in Equation (2) by taking into account all of the points in the two sets:

(3) $$\varepsilon_{\text{mean}}=\frac{1}{2}\left(\frac{1}{N_{1}}\sum_{i=1}^{N_{1}}\underset{{\text{q}}_{i}\in Q}{\min}\{d({\text{p}}_{i},{\text{q}}_{j})\}+\frac{1}{N_{2}}\sum_{j=1}^{N_{2}}\underset{{\text{p}}_{j}\in P}{\min}\{d({\text{p}}_{i},{\text{q}}_{j})\}\right)$$

**Figure 1.**
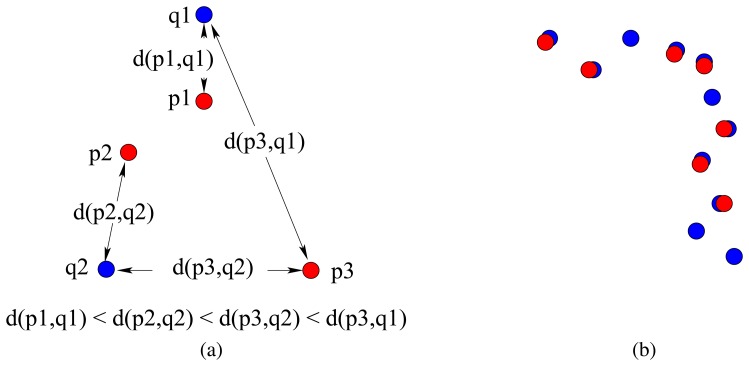
(a) Point set *P* in red, point set *Q* in blue and some of the Euclidean distances; (b) the case when *P* ⊂ *Q* and most of the error is contributed by the second term in Equation (3).

The distance of every point in set *P* to the nearest point in set *Q* is found and averaged, and vice versa. The two terms in Equation (3) are averaged so that the criterion is symmetric with respect to *P* and *Q*. For the simple example in Figure 1(a) where *N*_1_ = 3 and *N*_2_ = 2, the error is $\varepsilon_{\text{mean}}=\frac{d({\text{p}}_{1},{\text{q}}_{1})+d({\text{p}}_{2},{\text{q}}_{2})+d({\text{p}}_{3},{\text{q}}_{2})}{6}+\frac{d({\text{p}}_{1},{\text{q}}_{1})+d({\text{p}}_{2},{\text{q}}_{2})}{4}$. If the two sets of points are completely coincident, the average distance between the two sets will be zero. If one set is a subset of the other, there will be some error. Had an asymmetric criterion been employed, say including only the first (or the second) term in Equation (3), the error would have been zero when *P* ⊂ *Q* (or *Q* ⊂ *P*). The case when *P* ⊂ *Q* is illustrated in Figure 1(b) where the major contribution to the resulting error comes from the second term in Equation (3). Gaps occurring in the maps and sparsity are penalized by the error criterion, resulting in larger errors on the average.

The error criterion we propose is sufficiently general that it can be used to compare any two arbitrary sets of points with each other. This makes it possible to compare the accuracy of discrete point maps acquired with different techniques or sensing modalities with an absolute reference, as well as among themselves, both in 2-D and 3-D. When curves or shapes (e.g., lines, polynomials, snakes, spherical or elliptical caps) are fitted to the map points, the criterion proposed here also enables us to make an assessment of goodness of fit of the curve or shape to the map points. In other words, a fitted curve or shape comprised of a finite number of points can be treated in exactly the same way.

A widely used measure for comparing point sets in the computer vision area is the Hausdorff metric [27]. We included this metric in our study for comparison purposes. Hausdorff distance is commonly used for matching images in applications such as image analysis [28], visual robot navigation [29], target indexing in SAR images [30], computer-assisted surgery, etc. Basically, the Hausdorff metric is used to check if a template image is present in a test image so that the lower the value of the metric, the better is the match. It works well even in the presence of noise or occlusion of the target. Hausdorff distance can be defined as the maximum of the minimum distances of a set of points to the points in the other set.

More formally, the *directed Hausdorff distance* from set *P* to set *Q* is a *maximin* function, defined as:

(4) $$h(P,Q)=\underset{{\text{p}}_{i}\in P}{\min}\{\underset{{\text{q}}_{i}\in Q}{\min}\{d({\text{p}}_{i},{\text{q}}_{j})\}\}$$

where *d*(**p***_i_*, **q***_j_*) can, in fact, be any metric between these points. For simplicity, we take *d*(**p***_i_*, **q***_j_*) as the Euclidean distance between **p***_i_* and **q***_j_* as defined in Equation (1). Note that the Hausdorff distance, defined as above, is directed or asymmetric, meaning that most of the time, *h*(*P*, *Q*) ≠ *h*(*Q*, *P*). This asymmetry is a property of maximin functions, while minimin functions are symmetric.

A more general definition of Hausdorff distance is

(5) H(P,Q)=max{h(P,Q),h(Q,P)}

which defines the Hausdorff distance *between* sets *P* and *Q*, while Equation (4) defines the Hausdorff distance *from P to Q* (the directed Hausdorff distance). Referring to the example in Figure 1(a), *h*(*P*,*Q*) = *d*(**p**_3_, **q**_2_), *h*(*Q*, *P*) = *d*(**p**_2_, **q**_2_), and *H*(*P*, *Q*) = max{*d*(**p**_3_, **q**_2_), *d*(**p**_2_, **q**_2_) } = *d*(**p**_3_,q_2_) since *d*(**p**_3_, **q**_2_) > *d*(**p**_2_, **q**_2_).

Instead of taking the average or the maximum value among the minimum distance values, another possibility is to take the median of the minimum distances. In this case, a suitable error measure can be defined as:

(6) $$\varepsilon_{\text{median}}=\frac{1}{2}({\text{median}}_{{\text{p}}_{i}\in P}\left[\underset{{\text{q}}_{i}\in Q}{\min}\{d({\text{p}}_{i},{\text{p}}_{j})\}\right]+{\text{median}}_{{\text{q}}_{i}\in Q}\left[\underset{{\text{p}}_{i}\in P}{\min}\{d({\text{p}}_{i},{\text{q}}_{j})\}]\right)$$

Referring to our example in Figure 1(a), the median error is $\varepsilon_{\text{median}}=\frac{1}{2}\left[d({\text{p}}_{2},{\text{q}}_{2})+\frac{d({\text{p}}_{1},{\text{q}}_{1})+d({\text{p}}_{2},{\text{q}}_{2})}{2}\right]$. When there is an even number of minimum distances in the first or the second term of Equation (6), their median is found by averaging the two distances that fall in the middle.

For all of the criteria above, the two sets of points can be chosen as (i) two different sets of map points acquired with different mapping techniques or different sensing modalities (e.g., ultrasonic and laser, laser being the accurate reference), or (ii) two sets of fitted curve points (2-D) or shapes (3-D) (e.g., two polynomials, snake curves or spherical caps) to maps extracted by different mapping techniques or sensing modalities, or (iii) a set of extracted map points and a set of curve points fitted to them (i.e., assessment of goodness of fit).

## 3. Mapping Techniques

To demonstrate the use of the criterion through an example, we consider three ultrasonic mapping techniques. Most commonly, the large beamwidth of the transducer is accepted as a device limitation which determines the angular resolving power of the system. In this naive approach (method 1), a range reading of *r* from a transmitting/receiving transducer is taken to imply that the echo-producing object lies along the line-of-sight (LOS) of the transducer at the measured range. This is the simplest and the earliest approach, where a mark is placed along the LOS of the sensor at the measured range. It produces reasonable estimates for the positions of objects at nearby ranges or high frequencies of operation where the corresponding sensor beamwidth is small. If the sensor beamwidth is large, the angular position of the object is highly uncertain. Furthermore, this technique cannot eliminate any of the outliers or erroneous measurements.

The Arc-Transversal Median Algorithm (method 2) was developed by Choset and his co-workers, and requires both extensive bookkeeping and considerable amount of processing [18]. The algorithm can be summarized as follows: First, an ultrasonic arc map (UAM), representing the angular uncertainty of each range measurement is constructed. For each arc in the UAM, the positions of the intersection(s) with other arcs, if they exist, are recorded. For arcs without any intersections, the mid-point of the arc is taken to represent the actual point of reflection (as in method 1) which corresponds to the intersection point of the arc with the LOS. If the arc has a single intersection, the algorithm uses the intersection point as the location of the reflecting object. For arcs with more intersections, the median of the positions of the intersection points with other arcs is chosen to represent the actual point of reflection. In the work reported in [18], the median operation is applied when an arc has *three or more* intersection points. If there is an even number of intersections, the algorithm uses the mean of the two middle values (except that arcs with two intersections are ignored). It can be considered as a much improved version of method 1, where a single mark is placed along the LOS at the measured range.

Method 3 is based on directional processing of ultrasonic data and is detailed in [31]. This technique is based on the idea that in processing the acquired range data, there is a direction-of-interest (DOI) associated with each detected echo. Ideally, the DOI corresponds to the direction of a perpendicular line drawn from the sensor to the nearest surface from which an echo is detected. However, in practice, due to the angular uncertainty of the object position, the DOI can be approximated as the LOS of the sensor when an echo is detected. Since prior information on the environment is usually unavailable, the DOI needs to be updated while sensory data are being collected and processed on-line. It may also be possible to determine the DOI by post processing, based on the distribution of the acquired data by choosing it perpendicular to the direction along which the spread of the collected data over a given region is maximum. It should be noted that ideally, the DOI is different for each detected echo and sensor position.

The latter two methods are the most recently reported techniques for processing ultrasonic range measurements. All three methods use the same set of time-of-flight measurements acquired from the same environment. However, due to differences in processing the measurements, each one results in a different set of map points.

## 4. Experimental Procedure and Results

**Figure 2.**
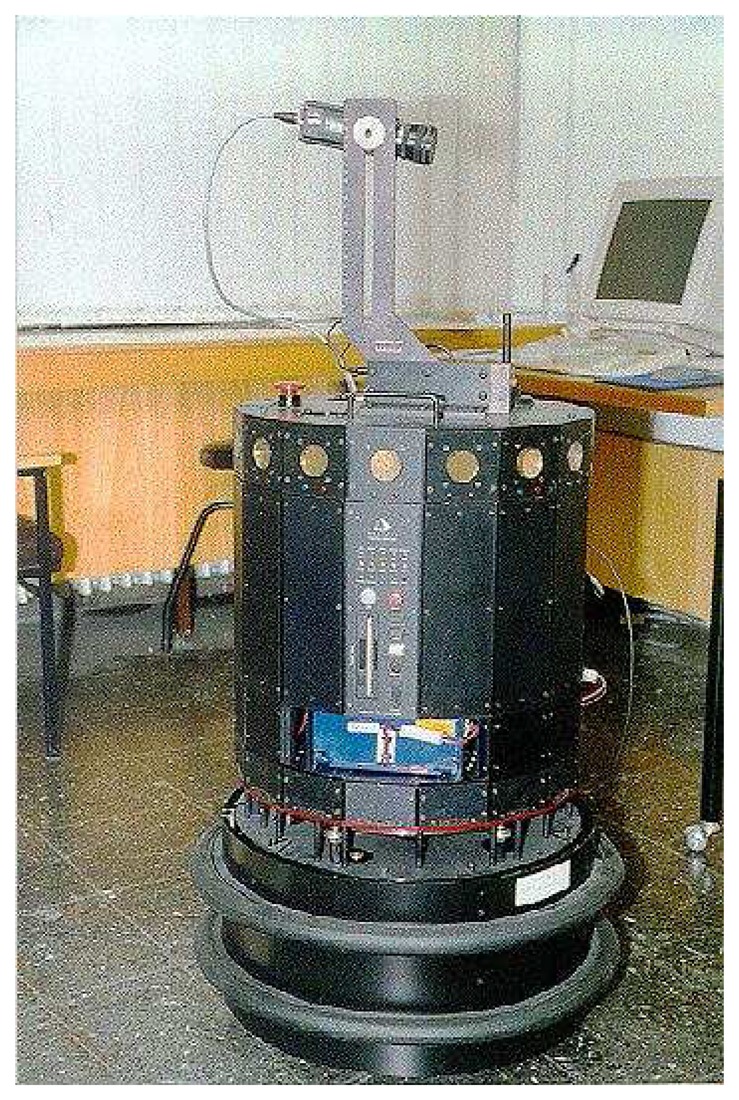
Nomad 200 mobile robot. The ring of ultrasonic sensors can be seen close to the top rim of the turret, and the structured-light laser system is seen pointing rightwards on top.

The experiments are performed using the Polaroid 6500 series ultrasonic transducers [32] available on the Nomad 200 mobile robot. The Nomad 200, shown in Figure 2, is an integrated mobile robot including tactile, infrared, ultrasonic, and structured-light sensing systems. The Polaroid 6500 has a resonance frequency of *f*_o_ = 49.4 kHz and aperture radius *a* = 2.0 cm, corresponding to a half-beamwidth angle of $\theta_{o}=\sin^{-1}\left[\frac{0.61_{c}}{af_{o}}\right]=12.2{}^{\circ}$. It measures time-of-flight *t*_o_, which is the round-trip travel time of the transmitted pulse between the transducer and the point of reflection, from which the range *r* can be calculated as *r* = *ct*_o_/2, where *c* is the speed of sound in air [33]. Results from three sample experiments are provided where ultrasonic time-of-flight and laser triangulation measurements are collected simultaneously. Both systems are rigidly fixed to the turret of the mobile robot so that the correspondence between them is never altered. The laser system is much more expensive, complex, and heavy, also requiring higher-power. Furthermore, this mode of sensing does not work in all environments, such as those with dark-colored upholstery or glass. Since it reveals a very accurate surface profile, the profile detected by the laser is taken as an absolute reference in the evaluation of the maps. In all of the experiments, the environment is divided into square elements or pixels of size 1 cm.

**Figure 3.**
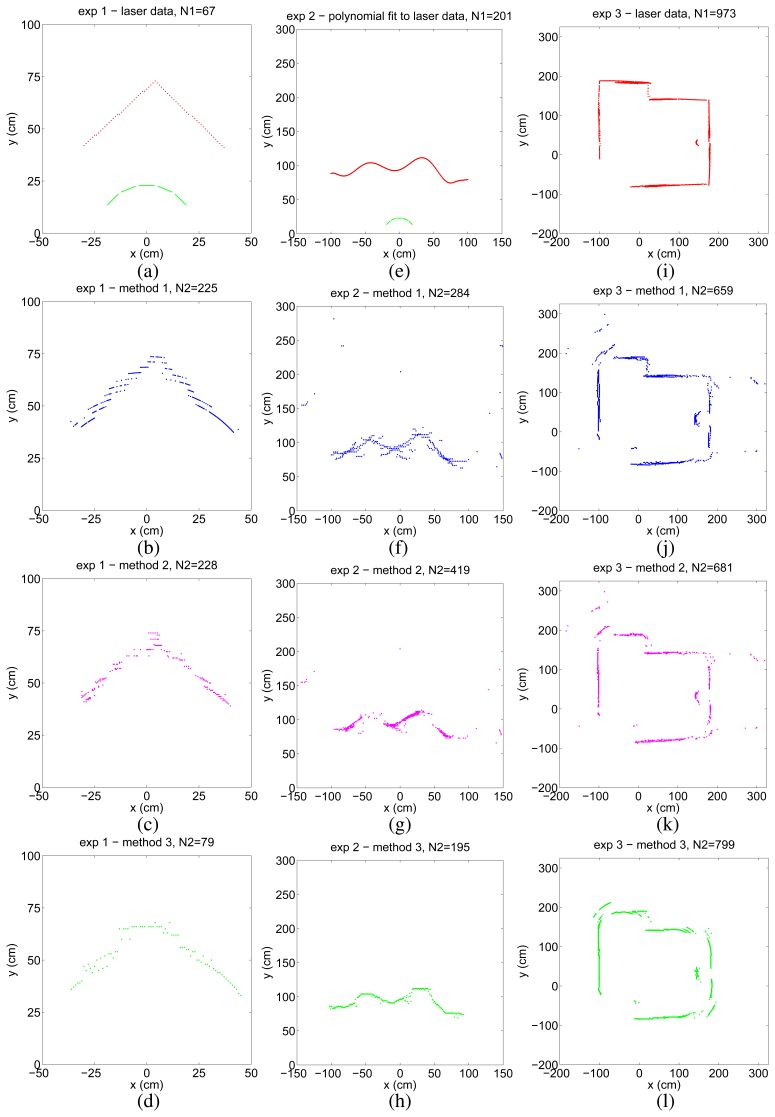
Laser data acquired from the three environments (top row, red) and the sensor configuration (top row, green); resulting maps of method 1 (2nd row, blue), method 2 (3rd row, magenta), and method 3 (4th row, green).

In the first experiment, we considered a 90° corner which is a typical feature of indoor environments. Due to its high curvature at the corner point, accurate mapping of this feature is usually difficult with ultrasonic sensors. The laser data obtained from the 90° corner and the locations of the five ultrasonic sensors are presented in Figure 3(a). Ultrasonic time-of-flight measurements were collected by rotating the ultrasonic sensors located on an arc from –30° to 30° in 1° steps. The results of the three mapping methods are shown in Figure 3(b)-(d).

**Figure 4.**
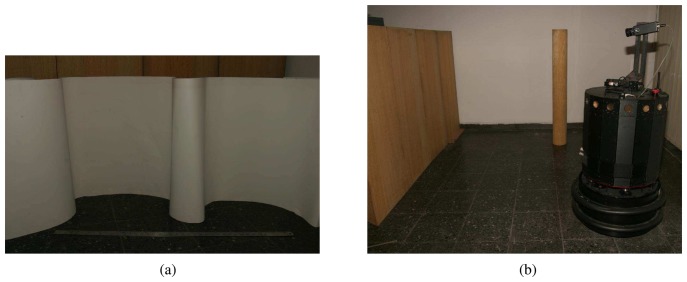
(a) An example curved surface and a meter stick; (b) a view of the indoor environment in Figure 3(i) looking towards the right, showing the top, right, and bottom walls.

For the second experiment, we have constructed curved surfaces of varying curvature and dimensions in our laboratory, using thin cardboard (see Figure 4(a) for an example). Again, only the front five ultrasonic sensors of the robot have been fired. Comparison is made with a 9th-order polynomial fit to the laser data (Figure 3(e)) obtained from one of the curved surfaces of height 1.05 m and length 3.65 m. In this experiment, the mobile robot simply translates along a straight path from (–75, 0) cm to (75, 0) cm alongside the surface at an average distance of 1 m and collects time-of-flight measurements by firing the front five ultrasonic transducers at every 2.5 cm. The turret is oriented such that both the laser and the ultrasonic sensors are directed towards the surface and it does not rotate throughout the translational movement. In the maps of methods 1 and 2 shown in Figure 3(f) and (g), there are some outlier points due to higher-order reflections, crosstalk, reflections from other objects in the environment, or totally erroneous measurements.

We also present experimental results from the indoor environment in Figure 4(b), comprising smooth wooden (left) and painted walls (middle) and a window shade (right) with vertical slats of 15 cm width. Some of the corners of the room are not perfect (e.g., where the shade and the painted wall make a corner on the right). There is a cylindrical object of radius 15 cm at a distance of 30 cm from the center of the painted wall. In this experiment, we use a simple rule-based wall-following algorithm for motion planning while collecting data. The mobile robot simply tries to follow the walls of the indoor environment trying to maintain a fixed distance to the walls, using sensory feedback from the front three sensors. The resulting laser map is illustrated in Figure 3(i) and the ultrasonic maps are shown in Figure 3(j)-(l). Again, the maps acquired with methods 1 and 2 contain many artifacts, especially exterior to the surrounded region in which the robot moves.

The errors are of the order of several pixels and are presented in Table 1. Obviously, the smaller the error, the better is the match between the ultrasonic map and the absolute reference. Overall, method 3 results in less artifacts and consequently smaller error in general, and is therefore considered to be superior to the other two methods. As we demonstrated with this example, the proposed error criterion allows us to make a quantitative comparison between the mapping results of different techniques or sensing modalities.

In Table 2, the directed Hausdorff distances *h*(*P*, *Q*) and *h*(*Q*, *P*) are tabulated for the three experiments where the set *P* corresponds to the resulting ultrasonic map points, and the set *Q* corresponds to the laser data. The Hausdorff distance *H*(*P*, *Q*) *between* sets *P* and *Q*, which is the larger of *h*(*P*, *Q*) and *h*(*Q*, *P*), is indicated by using boldface characters in the table. The Hausdorff distance results are much larger than *ε*_mean_ since this metric takes the maximum among the minimum values, whereas our proposed metric averages the minimum distance values. Consequently, the Hausdorff distance severely penalizes outlier points in the map. For this reason, method 3, which is successful at eliminating the outliers in the second and third experiments results in smaller errors in these experiments.

Since we are comparing ultrasonic map points with much more accurate laser data in this example, *h*(*P*, *Q*) is larger than *h*(*Q*, *P*) most of the time. This is explained by the fact that the points of ultrasonic maps are typically more distributed, fuzzy, and erroneous compared to laser maps so that the maximin distance *h*(*P*, *Q*) usually corresponds to the distance between one of the outliers of the ultrasonic map points and a laser data point. On the other hand, when *h*(*Q*, *P*) is calculated, there is usually an ultrasonic map point nearer to each laser data point than the outliers so that the effect of outliers on the error is reduced.

The results of calculating the median error are tabulated in Table 3. Note that most of the errors are smaller than the mean error *ε*_mean_ presented in Table 1, and all of the errors are much smaller than the Hausdorff distances given in Table 2. Since this approach can filter out the outlier points successfully, outlier map points are not penalized as much as in the first two approaches.

In summary, it can be concluded that the Hausdorff metric severely penalizes outlier measurements since it takes the maximum value among the minimum distances for the set points. The median error filters out the outliers and under-penalizes them because of the nonlinear filtering involved in taking the median. Our proposed metric provides a reasonable balance between these two metrics by weighting each of the minimum distances equally and calculating their average value bi-directionally.

## 5. Discussion

In this work, we have assumed that the robot or the sensing mechanism is well localized so that sufficiently accurate estimates of its pose are available. If this assumption is not true so that the localization errors are large, the acquired maps may be shifted and rotated with respect to the true map, resulting in increased error values. In this case, localization errors could be dominating the map error. If we are comparing simultaneously acquired maps not with the true map but among themselves, the relative error between the maps will not be much affected from the localization errors. Note that our criterion still remains applicable under these circumstances.

We have also assumed that the maps are acquired with the different sensing modalities under similar conditions. In the case of a dynamic environment, maps should be acquired simultaneously. For a static environment, the timing could be different. In the example that we have given, the two data sets were acquired simultaneously from the ultrasonic and structured-light laser systems on the mobile robot.

Another possibility that may result in large errors between the two point sets can occur when the two sensing modalities compared are so different in nature or at completely different positions in the environment that the corresponding views of the environment and the occluded or “shadowed” parts of the environment may be very different.

In case the sensing modalities take partial views or scans of the environment, we assume that they correspond to the same sectoral scan. Otherwise, the map points would have to be matched and their correspondence be found, which is beyond the scope of this work. It is worth emphasizing once again that in this work, we do not require the correspondence between the two sets of points to be known and do not make distinctions between the points of the same data set.

To summarize, the limiting circumstances for the proposed criterion could be caused by spatial or temporal differences in the acquirement of the two sets of map points.

## 6. Conclusion

We have presented an objective criterion to compare maps obtained with different techniques with an absolute reference as well as among themselves. This criterion can be used to compare two arbitrary sets of points with each other without requiring the correspondence between the two sets of points to be known. The two sets of points can correspond to: (i) two different sets of map points acquired with different mapping techniques or different sensing modalities, or (ii) two sets of fitted curve points to maps extracted by different mapping techniques or sensing modalities, or (iii) a set of extracted map points and a set of curve points fitted to them. We have demonstrated how the criterion can be used through an example from ultrasonic sensing based on time-of-flight measurements, considering accurate laser data as absolute reference and have compared the results with the Hausdorff metric and the median error results. We believe that the error criterion is sufficiently general so that it can be applied to both 2-D and 3-D range measurements of different scales, acquired with different mapping techniques and sensing modalities. Potential application areas for this work include millimeter wave radar for unmanned ground vehicles, mobile robotics, underwater sonar, optical sensing and metrology, remote sensing, ocean surface exploration, geophysical exploration, and acoustic microscopy. As part of the future work, it would be beneficial to demonstrate the applicability of the criterion to these sensing modalities at different scales.

## Figures and Tables

**Table 1.** Results of the three experiments based on the proposed criterion.

	*ε*_mean_ (pixels)		
	experiment 1	experiment 2	experiment 3

method 1	1.19	6.64	4.37

method 2	1.40	4.04	4.26

method 3	2.24	2.13	2.49

**Table 2.** Results of the three experiments based on the Hausdorff metric. The Hausdorff distance is highlighted in boldface fonts.

	Hausdorff distance (pixels)					
	experiment 1		experiment 2		experiment 3	

	*h*(*P*, *Q*)	*h*(*Q*, *P*)	*h*(*P*, *Q*)	*h*(*Q*, *P*)	*h*(*P*, *Q*)	*h*(*Q*, *P*)

method 1	**7.28**	2.24	**184.9**	6.08	**139.0**	16.12

method 2	**5.66**	3.16	**103.8**	8.54	**138.0**	17.00

method 3	**11.31**	5.00	8.94	**9.43**	**24.00**	14.00

**Table 3.** Results of the three experiments based on the median criterion.

	*ε*_median_ (pixels)		
	experiment 1	experiment 2	experiment 3

method 1	1.21	2.29	1.71

method 2	1.21	2.12	1.50

method 3	2.12	2.00	1.21
